# Supplementary material for: Global miRNA expression is temporally correlated with acute kidney injury in mice
Source: PeerJ. 2016 Feb 25;4:e1729. doi: 10.7717/peerj.1729 (PMC4782688; doi:10.7717/peerj.1729)
Supplement: Table S1 [file peerj-04-1729-s001.docx]

**Table S1.** Primer sequences of genes used in this study (mouse).

| Gene | Forward primer(5'-3') | Reverse primer (5'-3') |
| --- | --- | --- |
| miR-18a | GCGGCGGTAAGGTGCATCTAGTG | ATCCAGTGCAGGGTCCGAGG |
| miR-34b | GCGGCGGAATCACTAACTCCAC | ATCCAGTGCAGGGTCCGAGG |
| miR-134 | GCGGCGGTGTGACTGGTTGACC | ATCCAGTGCAGGGTCCGAGG |
| miR-182 | GCGGCGGTTTGGCAATGGTAGAAC | ATCCAGTGCAGGGTCCGAGG |
| miR-210 | GCGGCGGCTGTGCGTGTGACAG | ATCCAGTGCAGGGTCCGAGG |
| miR-214 | GCGGCGGACAGCAGGCACAGAC | ATCCAGTGCAGGGTCCGAGG |
| U6 | GCTTCGGCACATATACTAAAAT | CGCTTCACGAATTTGCGTGTCAT |
