# Supplementary material for: Global miRNA expression is temporally correlated with acute kidney injury in mice
Source: PeerJ. 2016 Feb 25;4:e1729. doi: 10.7717/peerj.1729 (PMC4782688; doi:10.7717/peerj.1729)
Supplement: Table S2 [file peerj-04-1729-s002.docx]

**Table S2.** Significantly over-represented KEGG pathways in renal pathology.

| KEGG term | Signaling pathway | False discovery rate |
| --- | --- | --- |
| mmu04310 | Wnt signaling pathway | 5.8×10^−5^ |
| mmu04115 | p53 signaling pathway | 7.6×10^−5^ |
| mmu04350 | TGF-β signaling pathway | 1.0×10^−4^ |
| mmu04630 | Jak-STAT signaling pathway | 4.7×10^−3^ |
| mmu04010 | MAPK signaling pathway | 9.4×10^−3^ |
| mmu04210 | Apoptosis | 1.2×10^−2^ |
| mmu04012 | ErbB signaling pathway | 1.2×10^−2^ |
| mmu04110 | Cell cycle | 1.3×10^−2^ |
| mmu04620 | Toll-like receptor signaling pathway | 1.6×10^−2^ |
| mmu04370 | VEGF signaling pathway | 4.2×10^−2^ |
