# Supplementary material for: Global miRNA expression is temporally correlated with acute kidney injury in mice
Source: PeerJ. 2016 Feb 25;4:e1729. doi: 10.7717/peerj.1729 (PMC4782688; doi:10.7717/peerj.1729)
Supplement: Table S3 [file peerj-04-1729-s003.docx]

**Table S3.** Renal pathology-related mRNAs targeted by miR-18a, -134, -182, -210, or -214.

| miRNA | Target gene | Full gene name |
| --- | --- | --- |
| mmu-miR-18a | pten | phosphatase and tensin homolog |
| mmu-miR-134 | limk1 | LIM-domain containing, protein kinase 1 |
| mmu-miR-134 | sox2 | SRY (sex determining region Y)-box 2 |
| mmu-miR-182 | chst1 | carbohydrate (keratan sulfate Gal-6) sulfotransferase 1 |
| mmu-miR-182 | fbxw7 | F-box and WD-40 domain protein 7 |
| mmu-miR-182 | ikzf1 | IKAROS family zinc finger 1 |
| mmu-miR-210 | bcl2 | B cell leukemia/lymphoma 2 |
| mmu-miR-210 | hif1a | hypoxia inducible factor 1, alpha subunit |
| mmu-miR-210 | shh | sonic hedgehog |
| mmu-miR-210 | tcf7l2 | transcription factor 7 like 2 |
| mmu-miR-210 | ucp2 | uncoupling protein 2 |
| mmu-miR-214 | pon2 | paraoxonase 2 |
